# Supplementary material for: What works, how and in which contexts when supporting parents to implement intensive speech and language therapy at home for children with speech sound disorder? A protocol for a realist review
Source: BMJ Open. 2024 Jan 6;14(1):e074272. doi: 10.1136/bmjopen-2023-074272 (PMC10773357; doi:10.1136/bmjopen-2023-074272)
Supplement: Supplementary data [file bmjopen-2023-074272supp001.pdf]

Appendix 1: Identification Screening Tool

	**Does the title/ abstract:**	Yes (2)	No (1)	Unsure (3)
1	Relate to a speech and language therapy intervention?	☐	☐	☐
2	Relate to intervention for primary speech sound disorder?	☐	☐	☐
3	Indicate intervention received by children aged 2-7 years?	☐	☐	☐
4	Relate to one of the following?			
	a. Involvement of parents in intervention	☐	☐	☐
	b. OR Online/ digital intervention	☐	☐	☐
	c. OR Intensity of intervention	☐	☐	☐
5	The population receiving intervention does not fall within the exclusion criteria outlined below:	☐	☐	☐
	If they do fall within the exclusion criteria, please specify which:	Autism Spectrum Disorder ☐<br>Down Syndrome ☐<br>Fragile X Syndrome ☐<br>Hearing impairment ☐<br>ADHD ☐<br>Cerebral Palsy ☐<br>Dysfluency ☐<br>Voice disorder ☐<br>Visual impairment ☐<br>Sensory processing difficulty ☐<br>Selective mutism		
6	To be included?	☐	☐	☐

Appendix 2: Selection Screening Tool

	**Does the paper:**	Yes (2)	Somewhat (1)	No (0)
1	Relate to speech and language therapy intervention?	☐	☐	☐
2	Relate to intervention for primary speech sound disorder?	☐	☐	☐
3	Relate to intervention for children aged 2-7 years?	☐	☐	☐
4	Relate to one of the following?			
	Involving parents in intervention?	☐	☐	☐
	OR Digital intervention?	☐	☐	☐
	OR Intensity of intervention?	☐	☐	☐
5	Describe the methodology used?	☐	☐	☐
		Outline methodology:		
6	Refer to or describe mechanisms or contexts of the intervention?	☐	☐	☐
	Overall score (out of 16)			
7	Relevance of study	High (2)	Moderate (1)	Low (0)
8	To be included?	Yes (2)	Possibly (1)	No (0)
		Reason:		

Relevance:

High (green):

- The research is very relevant to the realist review research questions.
- Contexts and mechanisms are clearly described.
- The paper adds rich insight to initial programme theories
- Makes specific reference to theory related to SSD (rather than SLCN)
- Children are predominantly within the age bracket of 2-7 years
- If looking at telehealth, the paper makes specific reference to theory that is relevant to asynchronous digital intervention
- If looking at intervention delivered by another person (not the SLT), then this is the child's parent/ carer.

Moderate (amber):

- The research has some relevance to the realist review questions and initial programme theories
- The research covers SSD in some way but may also describe intervention for other SLCN.
- Includes children aged 2-7 but this age is not the main focus of the study
- If looking at telehealth, the research may cover asynchronous intervention, but also looks at synchronous telehealth
- If looking at intervention delivered by another person, this may not be the parent/carer (e.g., school staff)

Low (red):

- The research has some relevance to the research question and initial programme theories, but may not offer rich insight

Appendix 3: Appraisal tool

	**Does the full text paper:**	Yes (2)	Somewhat (1)	No (0)
1	Help build on and/ or test (i.e., confirm, refine, refute) programme theories about:<br>Involving parents in intervention?<br>OR Digital intervention?<br>OR Intensity of intervention?	☐	☐	☐
2	Describe how the intervention was carried out?	☐	☐	☐
3	Give insight into mechanisms or contexts of the intervention?	☐	☐	☐
	Relevant comments			
4	Outline or refer to middle range theory related to:<br>Involving parents in intervention?<br>OR Digital intervention?<br>OR Intensity of intervention?<br><br>**Yes**: Clear overarching theory outlined.<br>**Somewhat:** Theory alluded to but not specified.<br>**No:** No links made to overarching theory.	☐	☐	☐
	Relevant comments			
5	Use methods that are credible, robust and trustworthy enough? Use the screening questions from MMAT/ PRISMA checklist/ SCRIBE.	☐	☐	☐
	Comments: Use screening questions from MMAT, SCRIBE or PRISMA. State strengths and weaknesses of the article. Any red flags?	Comments:		
6	Summary: Usefulness and relevance of study (please select):			
	High (2)	Moderate (1)	Low (1)	
	☐	☐	☐	
7	Summary: Rigour, credibility, and trustworthiness of study (please select):			
	High (2)	Moderate (1)	Low (1)	
	☐	☐	☐	
	Summary of the paper (brief overview of what it is about/ methods used/ type of data source etc.):			
8	Relevance score out of 10:			
9	Critical appraisal score:<br>MMAT (out of 5)<br>PRISMA checklist (out of 42)<br>SCRIBE (out of 26)			
10	Rigour score of data (out of 2):			
11	To be included?	Yes (2)	Possibly (1)	No (0)

Appendix 4: Data extraction tool

Author	
Year	
Objectives	
Research question	
Study type	
Study population (characteristics, inclusion or exclusion criteria)	
Age range of child receiving intervention	
Intervention focus (e.g., digital intervention, parent-based intervention, intensity of intervention)	
Programme description (location, timing, protocol, follow up etc.)	
Main outcomes	
Summary of methodological robustness and credibility (using previous appraisal tool)	
Key findings contributing to programme theory development:	
Mechanism	
Context (individual, interpersonal, infrastructural)	
Reference to middle-range theory:	
Identified by researcher	
Identified by author	
